# The Institutional Worker

**Published:** 1921-03-19

**Authors:** 


### THE INSTITUTIONAL WORKER.

*Being a Special Supplement to "The Hospital."*

### OUR BUREAU OF INFORMATION.

### INSTITUTION FACTS AND FIGURES.

### Hostels for Professional Women.

### THE HELENA RESIDENTIAL CLUBS.

We have more than once referred to the shortage of housing accommodation for women occupied on professional work in the large cities. This need is especially in evidence in London, where large numbers of women—engaged in work at such a distance from their homes as to preclude the possibility of returning to them each evening, or having no homes—are obliged to accept such accommodation as may be available. Only those who are acquainted with the conditions at present prevailing can appreciate the hardship which many are, in consequence, suffering. Because there is no choice in the matter, they must spend their leisure hours in surroundings which are quite unsuitable, and not infrequently detrimental to health; in some instances, indeed, opportunities for work are lost for the reason that housing, even of an indifferent character, cannot be found. In discussing various plans for tackling the problem and the success attendant on the scheme in operation at Washington of residential hotels for service women (see The Hospital Institutional Worker Supplement, January 8), we referred to the opportunity which the situation presented for co-operative enterprise in the establishment of residential clubs, which, from all the information that could be gleaned, seemed to provide exactly the kind of home needed by professional women.

Fortunately, the gravity of the situation has not been lost sight of altogether. Philanthropic men and women imbued with the spirit of personal service are grappling with the difficulty in a practical manner. An instance of this is the establishment of the Helena Residential Clubs, the first of which was opened at Lancaster Gate, Hyde Park, on November 12 last. Under the presidency of Princess Christian, who takes a keen and active interest in the work, the Club is constituted for business purposes as a limited company, the chairman of the directors being Sir C. Stewart, the late Public Trustee. While it is anticipated that the Club will pay its way—and there is no reason for doubting this, even to the payment of a moderate dividend on the capital expended—the question of profit did not enter into the consideration of the founders, who were actuated by a desire to do something toward the provision of a very urgent need, and to do it as quickly as possible.

It is interesting to note here that a certain portion of the capital was provided from the United Services Fund at the instance of the Women's Service Association, so that the Club might provide for Service women; and accordingly twenty-five beds are reserved for them. Candidates for membership are required to submit references to the House Committee, and to pay a subscription annually, which is at present limited to the moderate sum of 10s.

There is accommodation for forty-two residents, and the charges range from 30s. to 35s. a week for a cubicle or share of a bedroom. The charge includes breakfast and dinner each week-day, the addition of tea on Saturday and full board on Sunday. There are a few single rooms, for which a charge of £2 2s. a week, including board, is made. Beyond a small charge of 1d. (in the meter) for the use of gas-stove and gas-ring installed in each room, and of gas-irons provided in the laundry, there are no extras. Thus for a modest weekly sum the pleasure and comfort of a refined home may be enjoyed—a sum which is within the spending power of the many professional women whose incomes do not exceed £3 a week.

A visitor to the Club is at once impressed with the bright and happy atmosphere prevailing. It is not a club in the sense of the gloom and boredom of many such expensive institutions; it is a family of happy women in a well-appointed home. Compare it to the drab life in a dingy boarding-house, and who would not be awakened to the need for co-operative enterprise in this direction? The pleasing atmosphere results in a large degree from the personality of the superintendent, Miss McCall Anderson, who, after a distinguished hospital career, is devoting her administrative ability to this useful work. Miss McCall Anderson aims at making the Club as much like home as possible, and to this end she encourages the co-operation of the residents. There are no irksome restrictions. The door is locked at 10.30 p.m., but the residents are provided with latch-keys, and all that they need do when desiring to stay out later than that hour is to enter their names in a book provided in the hall.

The Club has an excellent dining-room, with a buffet, where meals are served at small tables; a bright and attractive lounge, a well-furnished "silence room," where residents may write or study in comfort, and a recreation-room. A bathroom and offices are provided on each floor, and a special feature is a small laundry, where small articles of clothing can be washed and ironed. Personal laundry is not, of course, catered for, and is sent out by the residents to such laundries as they select.

The cubicles are especially attractive; a typical example is a set of twelve arranged in a large and lofty room (originally the drawing-room). The partitions are partly wood frames with asbestos fillings, and partly curtains. Each cubicle is provided with a good bed, a metal cupboard fitted with trays, which also serves as a dressing-table; a looking-glass, and a metal wardrobe arranged at the foot of the cubicle. The whole presents an appearance of comfort combined with cleanliness. At one end of the room are arranged four wash-basins, with hot and cold water. There are also a gas-fire and gas-ring for boiling a kettle of water.

Those accustomed to the accommodation provided for nurses in an up-to-date hospital may not at first be well disposed toward the cubicle or share of a room. It is not, however, invariably possible to attain the ideal at once, and in these circumstances it is well to be content with the next best. As Miss McCall Anderson points out, it would be a practical impossibility to provide separate rooms in any but a house built for the purpose. The cost of building operations is almost prohibitive now, and the most important consideration must be to provide the best possible accommodation without delay. Moreover, the disadvantage of the cubicles is not so real as apparent, and the residents of the Club are genuinely satisfied with them.

It is hoped shortly to extend accommodation by equipping an adjoining house, which has already been leased for the purpose, and possibly to make provision also for non-resident members. Already the Club is full, and the waiting-list is daily lengthening.

### Question for March.

### WARD INVENTORIES.

**What is the simplest method of making an inventory of linen, crockery, furniture, etc., of wards and Nurses' Home? Give directions and examples, and state whether lists should be printed and hung in the wards and home.**

**(A minimum payment of 10s. 6d. will be made for each published answer.)**

RULES.

The following rules must be observed:—

1. Contributions must be written on one side of the paper. Conciseness and terseness are desirable features. The MS. must bear the name and address of the sender and be accompanied by coupon to be cut from the back cover (inside page) of the current issue of The Hospital. A pseudonym must be chosen if the name is not to be published.

2. Contributions must be addressed to the Editor of The Hospital Institutional Worker Supplement, 28 & 29 Southampton Street, Strand, London, W.C. 2, should reach him before the end of the current month, and be marked in the left-hand corner "Facts and Figures."

### QUESTIONS INVITED.

In connection with our Question Box, we offer every month five shillings for the best question sent in for consideration. The questions may be on any institutional subject and concern any department, from the secretary's to the porter's, or the matron's to the domestic staff; the one essential is that they must be practical and deal with points that have a definite relation to hospital or institutional administration, upkeep, finance, or management. Points relating to artificers' work, housekeeping, the laundry, out-patients, and other practical matters will be welcome. It is hoped that thus, when difficult or doubtful points arise in the course of their work, institutional workers will be encouraged to put them in the form of a question and send them to the Editor, The Hospital Bureau of Information, 28 Southampton Street, Strand, W.C. 2, marked "Question Box."

The best questions will be published in due course and our readers will be given the opportunity of answering them. Thus all institutional workers may be able to co-operate with the view of helping the work and smoothing the difficulties of each other. In short, we want the Question Box of the *Institutional Worker* to be freely used by every worker habitually.

### ENQUIRIES AND ANSWERS.

*THE SICK AND IN NEED.*

### Re-educating Crippled Lady.

We fear that we cannot suggest any home which would provide at once all the requirements. If no steps have yet been taken to obtain artificial limbs, this might occupy the first consideration, assuming that the patient's condition permits of it. Should the question of ways and means present a difficulty, application might be made to the Royal Surgical Aid Society, Salisbury Square, Fleet Street, E.C. 4. Inasmuch as the provision of limbs without education in their use is valueless, the opinion of the medical staff of the Society should be taken as to best means of obtaining the necessary education, as well as the type of apparatus suitable, having regard to the potential ability of the patient. It is possible that some conjoint arrangement could be made through the Society with the orthopædic department of a large general hospital, or with an orthopædic hospital, whereby the limbs and education in their use could be secured. Your first step, therefore, should be to submit a statement of the circumstances to the Secretary of the Surgical Aid Society, and to ask him to obtain for you advice upon the points in question. The doctor who has been attending the patient could perhaps do this most satisfactorily. Please write again if we can be of any further assistance.—R. E. H.

*EMPLOYMENT AND TRAINING.*

### Government Appointments Abroad.

Write for particulars to the Medical Department, India Office, Whitehall, S.W. 1; Secretary, Overseas Nursing Association, Imperial Institute, S.W.; Miss Ray, R.R.C., 54 Ashburnham Mansions, Chelsea, S.W. 10 (Lady Minto's Indian Nursing Association). We give you the names of some hospitals to which you might make application: Colombo General Hospital, Ceylon; British Seamen's Hospital, Smyrna; Campbell Hospital, 138 Lower Circular Road, Calcutta; Presidency General Hospital, 244 Lower Circular Road, Calcutta; Government Hospital, Upper Egypt, Assuan, Egypt; and the General Hospital, Shanghai. A full list of hospitals abroad is contained in "Burdett's Hospitals and Charities," which possibly the matron of your hospital keeps by her. We regret we cannot give postal replies; a coupon should be enclosed with each inquiry.—M. R.

### Private Nursing on Co-operative System.

Write to the Matron of the Royal Scottish Nursing Home for Private Patients, Moat Brae, Dumfries; also to the Assistant Matron of the Dumfries Branch of the Royal Scottish Nursing Institution, 14 Castle Street, Dumfries, and inquire if they have a co-operative branch. You will find a list of private nursing institutions in "How to Succeed as a Trained Nurse," published by the Scientific Press, Ltd., 28 and 29 Southampton Street, Strand, W.C. 2, price 3s. 3d. post free.—"Anxious Inquirer."

### Post Required.

If you cannot see a post such as you desire advertised in any of the nursing journals, perhaps it would be desirable for you to make your needs known in any one of the journals you mention. You might also apply to Messrs. Truman and Knightly, 158 Oxford Street, London, W. A coupon should be enclosed with each inquiry; we regret we cannot give postal replies.—J. J.

*MISCELLANEOUS.*

### Manufacturer of Silver Nit-Comb.

The silver nit-comb you inquire about can be obtained from the manufacturer, Mr. Geo. A. Binns, Archer Street Mills, Halifax.—"Matron."

### Catheterisation Prior to Operation.

It is desirable that the bladder should be empty prior to operation to which you refer. This is not invariably accomplished by the natural process; hence the use of the catheter. The cases to which you refer are doubtless female patients. If the usual precautions are observed and the instrument is handled with skill, no harm should result.—M. C.

### Iodine Spirit for Skin Preparation.

Industrial spirit in place of rectified spirit is just as efficient for making iodine spirit for preparation of the skin prior to operation, but the objection to it is that it causes excessive lachrymation, and many nurses who prepare patients with this solution suffer greatly from this. The cause is probably from gases given off by impurities in the methyl alcohol in combination with the iodine. Industrial spirit is so much cheaper than rectified spirit that there is a temptation to continue its use. It should, however, be borne in mind that nearly the whole of the duty on rectified spirit can be reclaimed by arrangement with the Excise officer, and the difference in the price is not, therefore, so large as it at first appears.—Inquirer.

### Handbook for Sister-Tutors.

We suggest that you obtain "The Theory and Practice of Nursing," by Miss M. A. Gullan, Sister-Tutor of St. Thomas's Hospital, obtainable of the Scientific Press, Ltd., 28 and 29 Southampton Street, Strand, London, W.C. 2, price 10s. 6d. net.—Sister.

### Irish Nurses' Association.

The Nurses' Club, Dublin, is now known as the Irish Nurses' Association; the address is 34 Stephen's Green, Dublin. The subscription of members is 2s. 6d. per annum.—Derry.

